# Draft Genome Sequence of Halophilic *Hahella* sp. Strain CCB-MM4, Isolated from Matang Mangrove Forest in Perak, Malaysia

**DOI:** 10.1128/genomeA.01147-17

**Published:** 2017-10-19

**Authors:** Ka-Kei Sam, Nyok-Sean Lau, Go Furusawa, Al-Ashraf Abdullah Amirul

**Affiliations:** a Centre for Chemical Biology, Universiti Sains Malaysia, Penang, Malaysia; b School of the Biological Sciences, Universiti Sains Malaysia, Penang, Malaysia

## Abstract

*Hahella* sp. strain CCB-MM4 is a halophilic bacterium isolated from estuarine mangrove sediment. The genome sequence of *Hahella* sp. CCB-MM4 provides insights into exopolysaccharide biosynthesis and the lifestyle of the bacterium thriving in a saline mangrove environment.

## GENOME ANNOUNCEMENT

*Hahella* sp. strain CCB-MM4 was isolated from soil samples obtained from Matang Mangrove Forest located in Perak, Malaysia, in September 2014 (1). The genus *Hahella* was first discovered in 2001; thus far, only three species that were isolated from a marine environment, namely, *Hahella chejuensis* (2), *Hahella ganghwensis* (3), and *Hahella antarctica* (4), have been described. Among them, the genome of *H. chejuensis* has been sequenced and published (5), while the genome of *H. ganghwensis* was registered in the NCBI database. Phylogenetic analysis revealed that strain CCB-MM4 is most closely related to *H. ganghwensis*, with a 16S rRNA gene sequence similarity of 98.43%, as analyzed using the EzTaxon server (6).

CCB-MM4 was cultured aerobically at 30°C with agitation in artificial seawater medium (7) until late-logarithmic phase, and the culture was used for genomic DNA extraction using the DNeasy blood and tissue kit (Qiagen, USA). A library for sequencing was constructed using the Nextera XT DNA sample preparation kit (Illumina, USA). The library was sequenced on an Illumina MiSeq instrument with 250-bp paired-end chemistry, and a sequencing coverage of more than 100-fold was obtained. *De novo* assembly of the reads was performed using SPAdes version 3.9.0 (8). The genome of *Hahella* sp. CCB-MM4 was assembled into 161 scaffolds, and the *N*_50_ value was 232,349 bp. The draft genome is 6,663,740 bp long, with an average G+C content of 49.7%. Genome annotation was accomplished using the Rapid Annotation using Subsystem Technology server and the NCBI Prokaryotic Genome Annotation Pipeline (9, 10). Annotation of the genome identified 6,110 predicted genes, of which 6,050 were protein-coding genes, and the remaining 60 were RNA genes (57 tRNAs and one 16S-23S-5S rRNA operon). Of the protein-coding genes predicted, 69.14% (4,184) were assigned a putative function and 30.84% were annotated as hypothetical proteins.

*H. chejuensis* is known for its ability to produce a large amount of extracellular polysaccharides (2, 5). Open reading frame prediction revealed the presence of genes related to exopolysaccharide synthesis in the genome of CCB-MM4. Genes encoding key enzymes involved in alginate biosynthesis, including mannose-1-phosphate guanylyltransferase, phosphomannomutase, and GDP-mannose 6-dehydrogenase, were detected in the genome. A group of genes responsible for alginate polymerization and secretion, *alg8*, *alg44*, *algE*, *algG*, *algK*, and *algL* (11), were also observed in the CCB-MM4 genome. The synthesis of extracellular polysaccharides enables bacteria to form biofilms and may confer an additional survival advantage in withstanding environmental stresses (12). Ectoine and hydroxyectoine biosynthesis is triggered in response to high or changing salinity, which enables halophiles to cope with the changes in osmotic pressure and survive under stress environments (13, 14). Ectoine and hydroxyectoine biosynthesis genes, including those for diaminobutyrate-2-oxoglutarate transaminase, l-2,4-diaminobutyric acid acetyltransferase, ectoine hydroxylase, and l-ectoine synthase, were annotated in the CCB-MM4 genome. Annotation also revealed that the genome of CCB-MM4 harbors genes encoding enzymes that are associated with cellulose hydrolysis (cellulase, β-glucanase, endoglucanase, and β-glucosidase) and chitin and *N*-acetylglucosamine utilization, as well as complex carbohydrate utilization genes. The genome data of *Hahella* sp. CCB-MM4 contribute to a better understanding of the biological potential of *Hahella* and will facilitate the uncovering of the industrial usefulness of this strain.

### Accession number(s).

This whole-genome shotgun project can be accessed at DDBJ/EMBL/GenBank under the accession no. MRYI00000000.
